# The association between self-rated health and risk of atrial fibrillation: a northern Sweden cohort study

**DOI:** 10.1016/j.pmedr.2026.103593

**Published:** 2026-07-29

**Authors:** Ebba Elofsson, Magdalena Johansson, Marcus M. Lind

**Affiliations:** Department of Public Health and Clinical Medicine, Umeå University, Umeå, Sweden

**Keywords:** Atrial fibrillation, Health status, Cohort study, Risk factor, Prospective study

## Abstract

**Objective:**

The aim of this study was to examine the association between self-rated health (SRH) and risk of atrial fibrillation (AFIB).

**Methods:**

We performed a cohort study including 104,198 middle-aged participants from the Västerbotten Intervention Programme, northern Sweden 1988–2014. All participants were free of AFIB at inclusion and completed a questionnaire including SRH. Participants were followed from the health examination to diagnosis of AFIB, death, migration or September 5, 2014. Cox regression analysis was used to estimate hazard ratios (HRs) with 95% confidence intervals (CIs).

**Results:**

Among participants, 26.6% rated their health as very good, 46.6 as good, 20.4% as average, 5.1% as somewhat poor, and 1.4% as poor. In total, 4775 participants were diagnosed with AFIB. In a multivariable model adjusted for cardiovascular risk factors, good (HR 1.13 [95% CI 1.04, 1.22]), average (HR 1.28 [95% CI 1.16, 1.40]), and somewhat poor (HR 1.26 [95% CI 1.09, 1.45]) SRH were associated with increased AFIB risk compared to very good SRH. *P*-value for trend for increased AFIB risk with worsening SRH was <0.01. A significant interaction between SRH and sex was observed.

**Conclusions:**

In conclusion, poorer SRH is associated with increased risk of first-time AFIB.

## Highlights

• We performed a cohort study of self-rated health and risk of atrial fibrillation.
• The study was performed in northern Sweden and included 104,198 participants.
• The risk of atrial fibrillation increased with lower self-rated health.
• The association remained significant after adjustment for potential confounders.

## 1 Introduction

Atrial fibrillation (AFIB) is the most common tachyarrhythmia affecting approximately 1–2% of the global population. The prevalence increases with advancing age (Kavousi, 2020). Among individuals above 75 years old, the rate reaches nearly 10%. From 1990 to 2019 the global prevalence of AFIB has doubled (Li et al., 2022). It is predicted that the prevalence and incidence will continue to increase due to aging populations and extended survival rates for chronic conditions (Kornej et al., 2020). Risk factors for AFIB include advanced age, male sex, European ancestry, smoking, diabetes mellitus, obesity and arterial hypertension (Staerk et al., 2017).

Recently, research has focused on treatment and complication management of AFIB, leading to advancements in e.g. anticoagulation therapy and rhythm control. Unfortunately, these improvements have not been matched by a proportional reduction in mortality (Calkins et al., 2017). Less attention has been given to prevent AFIB itself. To develop an effective preventive strategy, it is of importance identify high risk groups. Predictive scores, like the Framingham Heart Study Model (Schnabel et al., 2009) and CHARGE-AF (Goudis et al., 2023), might help identify those at higher risk. Biomarkers could also be used to predict AFIB (Filion et al., 2015; Shitole et al., 2024). However, these measures have not been widely established in clinical practice.

Self-rated health (SRH) refers to a person's personal evaluation of their overall well-being and health. Studies have highlighted the importance of SRH as a predictor of cardiovascular disease and mortality (Mavaddat et al., 2014). Exploring the potential association between SRH and the development of AFIB could lead to a simple and cost-effective approach for assessing AFIB risk in the general population. The association between SRH and the risk of future AFIB remains insufficiently explored. Lee et al. (2024) are the only researchers to date who have investigated this association. Their findings suggest that poor SRH is associated with an increased risk of developing AFIB. However, that study had a relatively small sample size, and did not provide sex-stratified analyses.

If an association between SRH and risk of AFIB is identified, SRH could potentially serve as an affordable and efficient complementary tool in the assessment of future AFIB risk. The identification of individuals at increased risk of developing AFIB may enable targeted preventive strategies such as lifestyles interventions.

The aim of this study was to investigate the association between SRH and risk of developing AFIB. We hypothesized that lower SRH would be associated with a higher risk of AFIB.

## 2 Methods

### 2.1 Study design and population

This study included participants from the Västerbotten Intervention Programme (VIP) conducted between January 1, 1988 and September 5, 2014. Inhabitants of Västerbotten County, northern Sweden, were invited to participate in a health examination, also including a questionnaire and lifestyle counselling, at ages 40, 50 and 60 years (initially also at 30 years of age) (Norberg et al., 2010; Späth et al., 2024). Individuals with a diagnosis of AFIB in the National Patient Register before the VIP were excluded. The basis for the present study was 109,697 participants of the VIP. Among these, 104,198 participants had data on SRH and were included. The participants were followed as a cohort from their health examination date until a diagnosis of AFIB, death, migration, or the end of study on September 5, 2014.

### 2.2 Measures

To investigate SRH, the following question in the VIP questionnaire was used: “What has your health been like during the last year?” with five possible responses: “Very good”, “Good”, “Average”, “Somewhat poor” and “Poor”.

Weight and height were measured, and body mass index (BMI) was calculated. Blood pressure was measured. Hypertension was defined as a systolic blood pressure ≥ 140 mmHg, a diastolic blood pressure of ≥90 mmHg or self-reported treatment with antihypertensives. Participants were defined as ever smokers or never smokers. Physical activity was categorized as low or high using leisure time physical activity at least once a week as a cut-off. Fasted blood samples were analysed for total cholesterol levels. Diabetes was determined based on self-reports, treatment with antidiabetics, a fasting plasma glucose ≥7.0 mmol/L, or a plasma glucose ≥12.2 mmol/L after a two-hour oral glucose tolerance test. Educational level was divided into maximum secondary school education and more than secondary school education. Data on history of myocardial infarction was derived from the VIP questionnaire. To define problem drinking, the Cut down, Annoyed, Guilt, Eye-opener (CAGE) questionnaire was used (Ewing, 1984). Two or more affirmative answers to the CAGE questionnaire was defined as problem drinking.

First-time AFIB events were identified by International Classification of Diseases (ICD) diagnosis codes in the National Patient register (Norberg et al., 2013). The codes used were ICD-10 code I48, ICD-9 code 427D and ICD-8 code 472.92.

### 2.3 Statistical analysis

Baseline characteristics were described using medians (1st and 3rd quartile boundaries) and numbers (%). To test for between-group differences, the chi-squared test, the Mann-Whitney *U* test and the Kruskal-Wallis test were used. Spearman's rank order test was used for correlation analyses.

The association between SRH and risk of AFIB was analysed using Cox proportional hazards regression. SRH was treated as a categorical variable. Hazard ratios (HRs) with 95% confidence intervals (CIs) and *p*-value for trend over categories of SRH were calculated. *P*-values <0.05 (two-sided) were considered significant. Analyses were performed for the entire cohort as well as for women and men separately. We performed univariable analyses, analyses adjusted for age and sex, and multivariable analyses adjusted for age, sex, BMI, hypertension, low physical activity, cholesterol, history of myocardial infarction, diabetes, education level and problem drinking.

A multivariable multiplicative interaction analysis was performed to explore the interaction between sex and SRH in the context of AFIB risk. We also performed multivariable additive interaction analyses to detect any interaction between SRH and hypertension and age (dichotomized at 45 years) respectively in relation to the risk of AFIB. As a sensitivity analysis, we included the health examination year in the multivariable model to account for changes over time. In another sensitivity analysis, we censored participants after 10 years of follow-up to minimize the risk of regression dilution bias (Clarke et al., 1999).

Participants in the study cohort with missing data were excluded from analyses encompassing that variable: BMI (*n* = 624), hypertension (*n* = 1048), ever smoker (*n* = 156), physical activity <1 time weekly (*n* = 2066), cholesterol (*n* = 748), diabetes (*n* = 601), maximum secondary school (*n* = 704), history of myocardial infarction (*n* = 919) and problem drinking (*n* = 3331).

A power calculation showed that we could detect a HR of 1.1 for increased risk of AFIB in the quartile of participants with the poorest SRH with 80% power and a significance level of 0.05 assuming a sample size of 100,000 participants. All analyses were performed using IBS SPSS Statistics for Macintosh, version 29.0.2 (IBM Corp., Armonk, NY, USA).

The study has been approved by the Regional Ethics Review Board, Umeå (approval number 2015–50-31 M, approved March 10, 2015). The study was conducted in accordance with the Declaration of Helsinki. Informed consent was obtained. All the institution's guidelines for protection of human subjects concerning their safety and privacy were met.

## 3 Results

### 3.1 Baseline characteristics

The VIP cohort included 104,198 participants with data on SRH. Among these, 53,912 were women (51%) and 51,286 (49%) were men. The median follow up-time was 14.8 years (range 0.0–25.2 years). At baseline, the median age was 42.5 years (Table 1). Of all participants, 49.9% had ever smoked, 71.7% had an education level below university level, 29.5% had diabetes and 7.1% had problem drinking. Among the participants, 26.6% rated their health as very good, 46.4% rated their health as good, 20.4% rated their health as average, 5.1% rated their health as somewhat poor and 1.4% rated their health as poor. Men rated their health higher than women (Table 1). Lower SRH was primarily reported by women, older participants, individuals with hypertension, smokers, and those with lower physical activity level, lower education, and higher alcohol consumption (Table 2). A Spearman's rank order correlation test showed that all adjustment variables were correlated with SRH (data not shown).

**Table 1:** Baseline characteristics of middle-aged northern Sweden Västerbotten Intervention Programme participants January 1, 1988 to September 5, 2014 with data on self-rated health and without a diagnosis of atrial fibrillation before health examination participation.

	All (n = 104,198)	Women (*n* = 52,912)	Men (*n* = 51,286)	P-value
Age, years	42.5 (40.0, 50.3)	40.8 (40.0, 50.3)	49.4 (40.0, 50.3)	0.01
Body mass index, kg/m^2^	25.2 (23.9, 28.0)	24.5 (22.2, 27.6)	25.8 (23.8, 28.3)	<0.01
Hypertension	30,474 (29.5)	13,679 (26.1)	16,795 (33.0)	<0.01
Ever smoker	51,940 (49.9)	26,225 (49.6)	25,715 (50.2)	0.05
Physical activity <1 time weekly	66,334 (64.9)	33,080 (63.8)	33,254 (66.1)	<0.01
Cholesterol, mmol/L	5.4 (4.7, 6.2)	5.3 (4.6, 6.1)	5.5 (4.8, 6.3)	<0.01
Diabetes	4373 (4.2)	1839 (3.5)	2534 (5.0)	<0.01
Maximum secondary school	74,172 (71.7)	35,426 (67.4)	38,746 (76.0)	<0.01
History of myocardial infarction	1052 (1.0)	220 (0.4)	832 (1.6)	<0.01
Problem drinking	7153 (7.1)	1784 (3.5)	5369 (10.8)	<0.01
Self-rated health				<0.01
Very good	27,684 (26.6)	13,541 (25.6)	14,143 (27.6)	
Good	48,345 (46.4)	24,082 (45.5)	24,263 (47.3)	
Average	21,301 (20.4)	11,155 (21.1)	10,146 (19.8)	
Somewhat poor	5363 (5.1)	3240 (6.1)	2123 (4.1)	
Poor	1505 (1.4)	894 (1.7)	611 (1.2)	

Characteristics are presented as medians (1st, 3rd quartile) for continuous variables and number (%) for categorical variables. P-values were obtained from the chi-squared test, the Mann-Whitney U test and the Kruskal-Wallis test.

**Table 2:** Baseline characteristics of middle-aged northern Sweden Västerbotten Intervention Programme participants January 1, 1988 to September 5, 2014 with data on self-rated health and without a diagnosis of atrial fibrillation before health examination participation. Data presented by level of self-rated health.

	Very good	Good	Average	Somewhat poor	Poor
Age, years	40.5 (40.0, 50.2)	40.6 (40.0, 50.3)	49.9 (40.1, 59.8)	49.9 (40.1, 59.8)	49.9 (40.1, 50.7)
Body mass index, kg/m^2^	24.6 (22.6, 27.0)	25.2 (23.0, 27.8)	26.0 (23.5, 29.0)	26.2 (23.4, 29.7)	26.3 (23.6, 29.8)
Hypertension	6326 (23.1)	13,749 (28.7)	7846 (37.2)	1997 (37.5)	556 (37.1)
Ever smoker	12,384 (44.8)	23,971 (49.6)	11,664 (54.9)	3035 (56.7)	886 (59.0)
Physical activity <1 time weekly	14,954 (55.0)	31,307 (65.9)	15,237 (73.4)	3788 (72.9)	1048 (72.7)
Cholesterol, mmol/L	5.4 (4.7, 6.1)	5.4 (4.7, 6.2)	5.5 (4.8, 6.3)	5.5 (4.8, 6.3)	5.5 (4.8, 6.4)
Diabetes	689 (2.5)	1778 (3.7)	1380 (6.5)	388 (7.3)	138 (9.2)
Maximum secondary school	18,349 (66.6)	34,520 (71.9)	16,137 (76.4)	4009 (75.3)	1157 (77.9)
History of myocardial infarction	116 (0.4)	341 (0.7)	423 (2.0)	131 (2.5)	41 (2.8)
Problem drinking	1351 (5.1)	3298 (7.0)	1884 (9.1)	492 (9.4)	128 (8.8)

Characteristics are presented as medians (1st, 3rd quartile) for continuous variables and number (%) for categorical variables.

### 3.2 SRH and the risk of AFIB

During the study period, 4775 cohort members (1776 women and 2999 men) were diagnosed with a first episode of AFIB. The overall incidence of AFIB in the study population was 3.46 cases per 1000 person-years. The AFIB incidence per 1000 person-years was 2.64 (1.55 in women; 3.77 in men) for those reporting very good SRH, 3.31 for good (2.41 in women; 4.26 in men), 4.62 for average (3.41 in women; 6.03 in men), 4.24 for somewhat poor (3.28 in women; 5.77 in men), and 4.18 for poor (3.27 in women; 5.61 in men).

The associations between SRH and risk of AFIB are presented in Table 3. In a univariable analysis including all study participants, having a SRH lower than very good was associated with an increased risk of AFIB compared to very good SRH. Individuals who rated their health as poor had an approximately 70% higher risk of AFIB (HR 1.72 [95% CI 1.36, 2.17]) in comparison to those who reported very good health. In a multivariable model, SRH ratings of good, average and somewhat poor were all associated with an increased risk of AFIB in comparison to the reference category of very good health. Participants with poor SRH did not have a significantly increased risk of AFIB. The *p*-value for trend was <0.01 across SRH categories in the multivariable analysis.

**Table 3:** The association between self-rated health and risk of atrial fibrillation in a cohort of middle-aged northern Sweden Västerbotten Intervention Programme participants January 1, 1988 to September 5, 2014 with data on self-rated health and without a diagnosis of atrial fibrillation before health examination participation.

	All			Women			Men		
	Univariable	Age/sex adjusted	Multivariable^a^	Univariable	Age adjusted	Multivariable^a^	Univariable	Age adjusted	Multivariable^a^
Age, per 10 years	2.91 (2.80, 3.02)	2.94 (2.84, 3.06)	2.69 (2.58, 2.81)	3.42 (3.21, 3.66)	3.42 (3.21, 3.66)	3.06 (2.82, 3.13)	2.71 (2.59, 2.83)	2.71 (2.59, 2.83)	2.54 (2.41, 2.67)
Male sex	1.90 (1.79, 2.02)	1.99 (1.88, 2.12)	1.96 (1.83, 2.09)	Stratified	Stratified	Stratified	Stratified	Stratified	Stratified
Body mass index, kg/m^2^	1.08 (1.08, 1.09)	1.06 (1.05, 1.07)	1.05 (1.04, 1.06)	1.08 (1.08, 1.09)	1.05 (1.04, 1.06)	1.04 (1.03, 1.05)	1.08 (1.07, 1.09)	1.06 (1.05, 1.07)	1.05 (1.04, 1.06)
Hypertension	2.91 (2.74, 3.08)	1.46 (1.38, 1.55)	1.30 (1.22, 1.39)	3.40 (3.09, 3.73)	1.51 (1.37, 1.67)	1.36 (1.21, 1.52)	2.51 (2.34, 2.70)	1.42 (1.32, 1.53)	1.27 (1.17, 1.38)
Ever smoker	1.18 (1.12, 1.25)	1.12 (1.06, 1.19)	1.11 (1.04, 1.18)	0.96 (0.87, 1.05)	1.19 (1.09, 1.31)	1.17 (1.05, 1.30)	1.32 (1.23, 1.42)	1.12 (1.04, 1.20)	1.11 (1.03, 1.21)
Physical activity <1 time weekly	1.23 (1.15, 1.31)	0.97 (0.91, 1.04)	0.90 (0.84, 0.97)	1.35 (1.21, 1.50)	1.04 (0.94, 1.16)	0.97 (0.86, 1.09)	1.14 (1.05, 1.23)	0.93 (0.86, 1.01)	0.88 (0.80, 0.95)
Cholesterol, mmol/L	1.19 (1.17, 1.22)	0.96 (0.93, 0.98)	0.95 (0.93, 0.98)	1.33 (1.29, 1.38)	0.95 (0.91, 0.99)	0.93 (0.90, 0.98)	1.09 (1.06, 1.12)	0.95 (0.92, 0.98)	0.95 (0.92, 0.98)
Diabetes	2.38 (2.14, 2.64)	1.37 (1.23, 1.52)	1.08 (0.96, 1.21)	2.65 (2.22, 3.16)	1.54 (1.29, 1.84)	1.26 (1.04, 1.54)	2.10 (1.84, 2.40)	1.29 (1.13, 1.47)	1.00 (0.86, 1.15)
Maximum secondary school	1.52 (1.41, 1.64)	1.09 (1.01, 1.18)	1.04 (0.96, 1.13)	1.91 (1.68, 2.17)	1.28 (1.12, 1.45)	1.20 (1.04, 1.38)	1.18 (1.07, 1.30)	0.98 (0.89, 1.08)	0.95 (0.85, 1.05)
History of myocardial infarction	4.30 (3.65, 5.07)	1.59 (1.35, 1.88)	1.33 (1.11, 1.59)	4.93 (3.32, 7.32)	2.23 (1.50, 3.32)	1.57 (1.01, 2.42)	3.38 (2.82, 4.05)	1.54 (1.28, 1.85)	1.33 (1.09, 1.61)
Problem drinking	1.24 (1.11, 1.38)	1.26 (1.13, 1.41)	1.21 (1.08, 1.36)	0.71 (0.50, 1.00)	1.02 (0.72, 1.45)	0.96 (0.66, 1.39)	1.07 (0.95, 1.20)	1.28 (1.14, 1.44)	1.24 (1.10, 1.40)
Self-rated health									
Very good	1 (ref.)	1 (ref.)	1 (ref.)	1 (ref.)	1 (ref.)	1 (ref.)	1 (ref.)	1 (ref.)	1 (ref.)
Good	1.28 (1.18, 1.38)	1.21 (1.12, 1.30)	1.13 (1.04, 1.22)	1.61 (1.40, 1.84)	1.41 (1.23, 1.61)	1.27 (1.10, 1.47)	1.14 (1.04, 1.25)	1.11 (1.02, 1.22)	1.06 (0.96, 1.17)
Average	1.82 (1.68, 1.98)	1.44 (1.33, 1.57)	1.28 (1.16, 1.40)	2.34 (2.03, 2.70)	1.66 (1.43, 1.91)	1.39 (1.19, 1.63)	1.65 (1.49, 1.83)	1.34 (1.21, 1.49)	1.23 (1.10, 1.38)
Somewhat poor	1.73 (1.51, 1.97)	1.46 (1.28, 1.66)	1.26 (1.09, 1.45)	2.35 (1.92, 2.89)	1.77 (1.44, 2.17)	1.55 (1.24, 1.93)	1.62 (1.36, 1.93)	1.31 (1.10, 1.56)	1.11 (0.92, 1.35)
Poor	1.72 (1.36, 2.17)	1.51 (1.20, 1.91)	1.21 (0.93, 1.57)	2.35 (1.66, 3.32)	1.81 (1.28, 2.57)	1.50 (1.03, 2.20)	1.61 (1.17, 2.23)	1.36 (0.99, 1.88)	1.06 (0.74, 1.52)
*P-value over trend for categories of self-rated health*	<0.01	<0.01	<0.01	<0.01	<0.01	<0.01	<0.01	<0.01	<0.01

Associations are shown as hazard ratios with 95% confidence intervals. *P*-values over trend were obtained from Cox proportional hazards regression models.

a Multivariable model adjusted for age, sex, body mass index, hypertension, low physical activity, cholesterol, history of myocardial infarction, diabetes, education level and problem drinking.

**Fig. 1:**
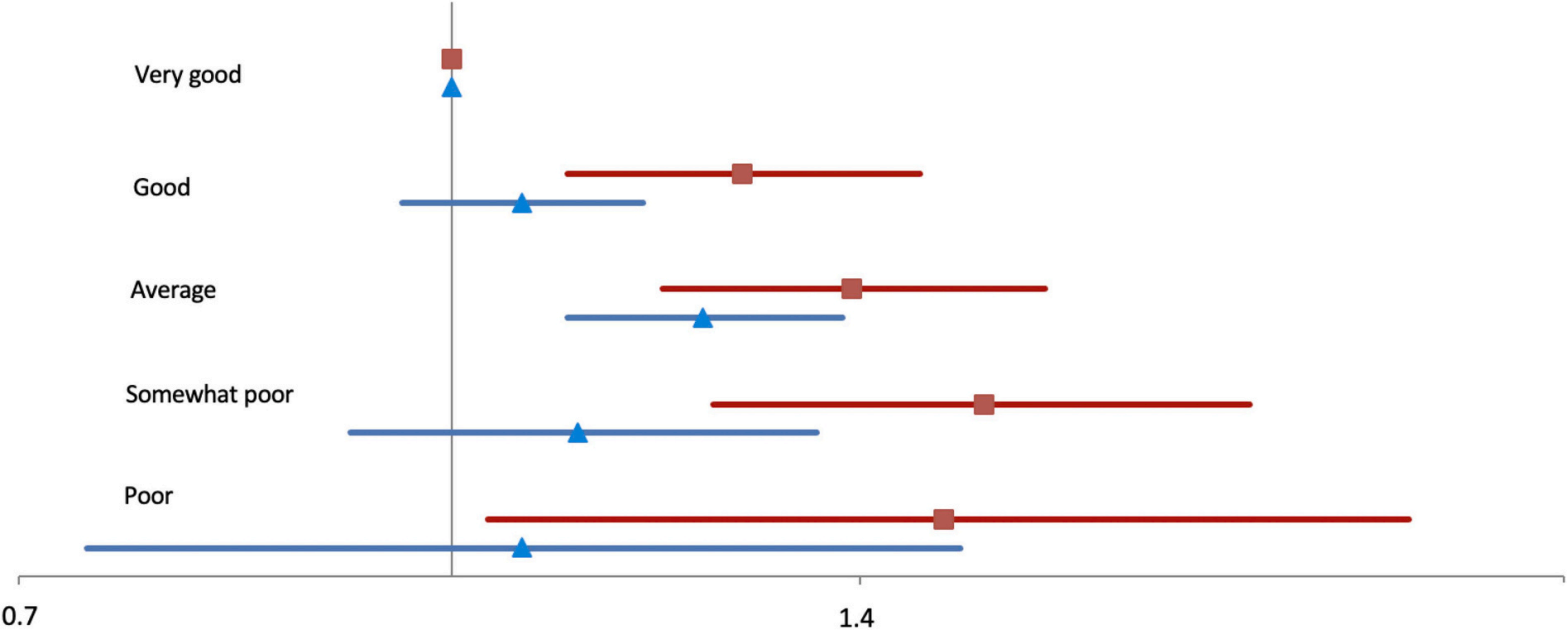
The association between self-rated health and risk of atrial fibrillation in women and men in a cohort of middle-aged northern Sweden Västerbotten Intervention Programme participants January 1, 1988 to September 5, 2014 with data on self-rated health and without a diagnosis of atrial fibrillation before health examination participation. The association between self-rated health and risk of atrial fibrillation in women and men. The multivariable association between self-rated health and risk of atrial fibrillation in women (red squares) and men (blue triangles) are shown as hazard ratios with 95% confidence intervals. The multivariable model was adjusted for age, sex, body mass index, hypertension, low physical activity, cholesterol, history of myocardial infarction, diabetes, education level and problem drinking. (For interpretation of the references to color in this figure legend, the reader is referred to the web version of this article.)

A multiplicative interaction analysis revealed an interaction between sex and SRH in the context of AFIB risk, with a p-value of 0.03. Then, data was analysed for women and men separately (Table 3 and Fig. 1). In a univariable model, both women and men who assessed their health at any level below very good had a significantly increased risk of AFIB with numerically higher HRs in women. The P for trend over worsening SRH was <0.01 in both men and women. In a multivariable model, compared with very good SRH, all categories of poorer SRH were significantly associated with increased risk of AFIB in women. In men, good SRH was the only category significantly associated with an increased risk of AFIB compared to very good SRH.

Multivariable additive interaction analyses were performed to detect potential interactions between SRH and hypertension and age respectively in relation to risk of AFIB. The additive interaction analyses indicated an interaction between SRH and age in relation to risk of AFIB. SRH seemed to be a more important predictor of AFIB in participants aged 45 years and up at baseline. No apparent additive interaction was found between SRH and hypertension. In a sensitivity analysis, participants were censored after 10 years of follow-up. Another sensitivity analysis was performed with adjustment for year of health examination in the multivariable model. In both sensitivity analyses, HRs for associations between SRH and risk of AFIB were similar to those of the main analysis (data not shown).

## 4 Discussion

We found that poorer SRH was associated with an increased risk of first-time AFIB in both women and men. Additionally, we found a significant interaction between sex and SRH in relation to AFIB risk.

Our results show partial agreement with those reported in a prior study by Lee et al. (2024). In their study, a significant association between SRH and AFIB was observed after adjusting for potential confounders. However, sex-stratified analyses were not presented, possibly due to the smaller sample size and fewer AFIB events compared to our study. Another difference is that our study used a five-point scale to assess SRH, whereas their study used a three-point scale. A greater number of response options may provide more nuanced information on perceived health. Notably, our study validates their findings and establishes the association between SRH and risk of AFIB. Furthermore, our sex-stratified analysis adds to the understanding of this association.

SRH has also been studied in relation to other cardiovascular conditions, Waller et al. (2015)) found an association between SRH and increased risk of myocardial infarction in a prospective cohort study. In contrast to our study, no interaction between SRH and sex was observed. On the other hand, in a study by Nymberg et al. (2022) concerning the association between SRH and venous thromboembolism, an interaction between sex and SRH was noted. Good SRH was associated with a reduced risk of venous thromboembolism in women, while no such association was observed in men. A study by Nylund et al. (2023) also indicated possible differences between men and women in the association between SRH and risk of venous thromboembolism although no interaction analysis was made. Additionally, a previous study reported that the validity of SRH was greater among women than among men (Baćak and Ólafsdóttir, 2017).

Potential underlying factors explaining the observed association between poor SRH and increased risk of AFIB may include the presence of health conditions that are established risk factors for AFIB and at the same time contribute to poor SRH. This hypothesis is strengthened by the fact that the associations seen in the univariable and the age- and sex adjusted models were partially attenuated in the multivariable model. However, the results were still statistically significant. It is possible that additional adjustment for other health conditions would have attenuated the results further.

Additionally, poor SRH might induce psychological stress, which could potentially increase the probability of developing AFIB in the future. Furthermore, individuals reporting poor SRH may also have a reduced capacity or opportunity to engage in health-promoting behaviours that could be protective against AFIB. It is important to note, however, that the present study was not designed to investigate the causal mechanisms linking SRH to subsequent AFIB risk. As demonstrated in our results, substantial differences exist between individuals with varying levels of SRH in terms of lifestyle factors and comorbidities.

A strength of the present study is the population-based design which increases the external validity. There were only small differences between participants and non-participants of the VIP, and the attendance rate was high (approximately 60%) (Norberg et al., 2012). Another strength is the large number of participants with a balanced number of women and men which allowed for stratified analyses. The access to health examination data enabled us to adjust for a wide range of confounders. Our outcome assessment using the National Patient Register has been shown to be a good way of identifying AFIB cases in Sweden. A diagnosis of AFIB in the National Patient Register has a positive predictive value of 95.5% (Norberg et al., 2013). We estimate that this method captured a vast majority of symptomatic incident AFIB cases in our study population.

A limitation of our study is the potential presence of unmeasured confounders that may vary across SRH groups. It is possible that such unidentified factors could have influenced our findings. It is plausible that poor SRH and AFIB share common underlying risk factors. However, the aim of this study was not to establish a causal relationship between SRH and AFIB risk, but rather to explore the potential of SRH as a tool to predict the risk of AFIB.

The definition of AFIB used in the present study was based on ICD diagnosis codes. Thus, only participants that had received a diagnosis of AFIB by a medical professional were captured as cases. Furthermore, our definition of AFIB was dichotomous and did not account for the percentage of time spent in AFIB. This is a limitation as the AFIB burden, defined as the proportion of time spent in AFIB, is an important measure relevant for e.g. the symptoms and outcomes related to AFIB (Doehner et al., 2025).

The growing use of wearables that can detect episodes of AFIB may lead to an increase in the number of persons with known AFIB (Chan et al., 2025). Possibly, the predisposition to use such wearables could vary between persons with different levels of SRH, thus leading to differences in AFIB detection rates.

Adjustments were made for low physical activity level due to its probable link to poorer SRH, but strenuous physical activity was not considered. Practicing strenuous physical activity is likely to be positively associated with SRH and seems to be associated with an increased risk of AFIB in young and middle-aged men (Guasch and Nattel, 2021). An example is AFIB occurring in individuals practicing strenuous endurance exercise (Sanchis-Gomar et al., 2017). An experimental animal study showed that AFIB inducibility increased with exercise load and that intensive exercise led to increased atrial fibrosis, to enriched inflammatory pathways in the right atrium and to a transient increased in proinflammatory plasma biomarkers (Alcarraz et al., 2025). Including data on strenuous physical activity could be considered in future studies of the association between SRH and AFIB risk.

Another potential limitation of this study is the long follow-up time which introduces risk of regression dilution bias. We have tried to account for this by restricting the follow-up time to ten years. As this did not substantially alter our results, we think that the regression dilution bias does not affect our results to a large degree.

The generalizability of our results to other populations might be limited as most study participants were of Western European origin. It has been previously demonstrated that both self-perceived health status and the prevalence of AFIB vary across ethnic groups (Bombak and Bruce, 2012; Ugowe et al., 2018). Moreover, variations in the incidence of AFIB and its associated risk factors are also observed among different age groups (Bizhanov et al., 2023). Therefore, the generalizability of our results to age groups beyond those included remains uncertain.

## 5 Conclusion

In conclusion, we found that poorer SRH was associated with an increased risk of first-time AFIB in both middle-aged women and men. Also, our study showed a significant interaction between sex and SRH in relation to risk of AFIB. Notably, our findings underline that SRH is an easily assessable measure that could be a useful addition to established risk factors in the process of identifying individuals at increased risk of future AFIB.

## Declaration of generative AI and AI-assisted technologies in the manuscript preparation

During the preparation of this work, the authors used ChatGPT (GPT-4o, 2024) in order to improve the use of the English language in the manuscript. After using this tool, the authors reviewed and edited the content as needed and take full responsibility of the content of the published article.

## CRediT authorship contribution statement

**Ebba Elofsson:** Writing – original draft, Visualization, Formal analysis. **Magdalena Johansson:** Writing – review & editing, Supervision, Investigation, Funding acquisition, Data curation, Conceptualization. **Marcus M. Lind:** Writing – review & editing, Supervision, Investigation, Funding acquisition, Conceptualization.

## Funding sources

This work was supported by the 10.13039/501100002915Foundation for Medical Research in Skellefteå and 10.13039/501100002960Västerbotten County Council.

## Declaration of competing interest

The authors declare that they have no known competing financial interests or personal relationships that could have appeared to influence the work reported in this paper.

## Data Availability

The authors do not have permission to share data.
